# Unveiling Global Diversity of Patescibacteriota and Functional Interactions with Host Microbes

**DOI:** 10.1002/advs.202509416

**Published:** 2025-12-12

**Authors:** Yanhan Ji, Xu Liu, Shuai Zhao, Sihan Zhou, Yufan Yang, Ping Zhang, Yu Shi, Wei Qin, Guibing Zhu, Yongguan Zhu, Yanzheng Gao, Jiandong Jiang, Baozhan Wang

**Affiliations:** ^1^ Key Laboratory of Agricultural and Environmental Microbiology Ministry of Agriculture and Rural Affairs Department of Microbiology College of Life Sciences Nanjing Agricultural University Nanjing Jiangsu 210014 China; ^2^ State Key Laboratory of Desert and Oasis Ecology Xinjiang Institute of Ecology and Geography Chinese Academy of Sciences Urumqi 830011 China; ^3^ State Key Laboratory of Herbage Improvement and Grassland Agro‐ecosystems Centre for Grassland Microbiome, College of Pastoral Agriculture Science and Technology Lanzhou University Lanzhou Gansu 730020 China; ^4^ State Key Laboratory of Crop Stress Adaptation and Improvement School of Life Sciences Henan University Kaifeng 475004 China; ^5^ School of Biological Sciences and Institute for Environmental Genomics University of Oklahoma Norman OK 73072 USA; ^6^ Research Center for Eco‐Environmental Sciences Chinese Academy of Sciences Beijing 100085 China; ^7^ State Key Laboratory of Urban and Regional Ecology Research Centre for Eco‐Environmental Sciences Chinese Academy of Sciences Beijing 100085 China

**Keywords:** CPR, episymbionts, metabolic complementation, nitrite detoxification, patescibacteriota, ribosomal protein S3

## Abstract

Patescibacteriota, also known as Candidate Phyla Radiation (CPR), is a diverse clade of ultra‐small bacteria with an epibiotic lifestyle. Despite their ubiquity across diverse ecosystems and ecological importance in microbial networks, the global distribution of Patescibacteriota and functional interactions with their host organisms remain largely unknown. Here, by leveraging comprehensive Patescibacteriota genomic resources and global multi‐habitat metagenomic datasets, it is demonstrated that ribosomal protein S3 (rpS3) as a reliable phylogenetic marker, enabling accurate recovery of Patescibacteriota diversity from short‐read metagenomes. Using this framework, extensive taxonomic diversity and pronounced community heterogeneity are uncovered across eight ecosystems. Through network analysis and genome‐wide functional screening, habitat‐specific co‐occurrence patterns are also revealed between Patescibacteriota and host‐associated bacteria, especially potential functional synergies mediated by metabolic pathway cascades. Notably, Patescibacteriota‐encoded NirK may assist sulfate‐reducing bacteria in resisting nitrite stress, while NorB can mitigate nitric oxide toxicity for complete ammonia‐oxidizing bacteria. Taken together, this study highlights the underappreciated diversity of Patescibacteriota and elucidates its important role in supporting host metabolism through complementary biochemical functions, offering new insights into its ecological significance and evolutionary adaptations in the global ecosystem.

## Graphical abstract

Patescibacteriota represents a diverse group of ultra‐small epibiotic bacteria, which is largely overlooked. By integrating ribosomal protein S3‐based community profiling with MAG‐based metabolic potential analyses, this study provides new insights into their distribution, diversity, and potential interactions with other bacteria across diverse metagenomes.

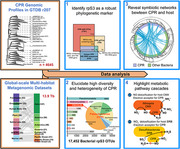

## 1 Introduction

Patescibacteriota, now recognized in the Genome Taxonomy Database (GTDB) as a single phylum, represents one of the most enigmatic yet pervasive groups of microbial life on Earth.^[^ ^1^, ^2^, ^3^, ^4^ ^]^ Historically, this lineage was described as the Candidate Phyla Radiation (CPR), originally thought to comprise more than 74 separate candidate phyla based on a 16S rRNA gene sequence identity threshold, which have since been unified under the phylum Patescibacteriota in GTDB.^[^ ^1^, ^4^ ^]^ These microorganisms share two hallmark features: ultrasmall cell sizes (typically < 0.01 µm^3^) and drastically reduced genomes (often < 1.5 Mbp) that lack essential biosynthetic pathways.^[^ ^1^, ^4^, ^5^ ^]^ Such extreme genome reduction challenges conventional definitions of bacterial autonomy, suggesting lifestyles distinct from those of free‐living microbes. This paradox of high diversity coupled with minimal metabolic capacity finds resolution in their epibiotic lifestyle.^[^ ^6^, ^7^, ^8^, ^9^ ^]^ Recent reports demonstrate that this expansive taxon may constitute over 15% of bacterial diversity, broadening the understanding in biodiversity of microbial dark matter.^[^ ^1^, ^10^ ^]^ These traits confer dual ecological importance as repositories of unexplored microbial diversity and as critical interacting network members in complex microbial systems.^[^ ^11^ ^]^ Nevertheless, while their host‐dependent ecology implies tight biogeographic coupling, the global distribution patterns of Patescibacteriota remain elusive, posing a fundamental gap in understanding microbial biodiversity and interactions in a changing world.

Patescibacteriota have been documented across a wide range of biomes—including aquatic environments,^[^ ^7^, ^12^, ^13^, ^14^, ^15^ ^]^ terrestrial ecosystems,^[^ ^16^, ^17^ ^]^ host‐associated microbiomes,^[^ ^17^, ^18^, ^19^ ^]^ and extreme habitats^[^ ^20^, ^21^ ^]^—yet methodological constraints to limit a comprehensive understanding of their ecological dynamics. Amplicon sequencing is severely affected by primer biases and insertions within the 16S rRNA gene, leaving more than 70% of some Patescibacteriota lineages undetected and thus grossly underestimating their diversity.^[^ ^1^, ^22^ ^]^ Although PCR‐independent metagenomic sequencing has improved taxonomic recovery, reconstruction of 16S rRNA genes and metagenome assembled genomes (MAGs) from low‐abundance taxa frequently yields incomplete assemblies, distorting both diversity estimates and functional inference.^[^ ^15^, ^23^, ^24^ ^]^ More recently, ribosomal protein S3 (rpS3)‐based community profiling derived from metagenomic scaffolds has been increasingly applied to investigate the diversity and community composition of Patescibacteriota—and even prokaryotes more broadly—within metagenomic datasets.^[^ ^23^, ^25^, ^26^, ^27^ ^]^ Thus, the integration of rpS3‐based community profiling with MAG‐based metabolic potential analyses emerges as a plausible strategy for advancing our understanding of Patescibacteriota diversity and their host‐linked metabolic roles across ecosystems.

The ecological significance of Patescibacteriota is fundamentally rooted in their obligate physical associations with host organisms, with experimental evidence showing that direct attachment is indispensable for their growth and survival.^[^ ^9^, ^28^ ^]^ Such intimate interactions create microscale niches that facilitate metabolic exchange, protein modification, and other molecular interactions, thereby exerting profound effects on host physiology and function. A well‐studied example is the TM7 lineage in the human oral microbiome, which encodes the arginine deiminase system to enhance host tolerance to acidic environments and, as demonstrated in murine models, mitigates inflammation by modulating host pathogenicity.^[^ ^29^, ^30^ ^]^ Moreover, certain TM7 strains dynamically regulate phage receptor expression, mediating host‐phage equilibria and stabilizing microbial communities against viral predation.^[^ ^31^ ^]^ Despite these insights, mechanistic understanding remains extremely limited: all experimentally validated Patescibacteriota‐host interactions to date stem from fewer than 0.1% of cultivable strains, leaving the vast majority of lineages functionally uncharacterized. This narrow evidence base obscures three fundamental questions: (i) the phylogenetic breadth of Patescibacteriota‐mediated community regulation, (ii) the habitat plasticity of interaction networks, and (iii) the evolutionary principles underpinning Patescibacteriota‐host co‐diversification. Together, these knowledge gaps severely constrain predictive models of microbiome dynamics and highlight the urgent need for integrative ecological and experimental frameworks.

To address these critical knowledge gaps, we applied an integrated multi‐omics framework encompassing 4645 Patescibacteriota MAGs and 13.9 terabases (Tb) of metagenomic data spanning eight major biomes, including freshwater, marine, terrestrial, and engineered systems. This large‐scale dataset enabled a systematic evaluation of Patescibacteriota diversity and ecological contributions. We show that rpS3 functions as a robust phylogenetic marker capable of accurately delineating Patescibacteriota diversity within short‐read metagenomes, and a standardized rpS3 reference database anchored to GTDB R207 taxonomy was established to support future studies. Our analyses reveal that Patescibacteriota constitutes one of the most diverse bacterial phyla globally and displays pronounced community heterogeneity across ecosystems. Network analyses further uncover habitat‐specific co‐occurrence patterns between Patescibacteriota and their potential hosts, while genome‐resolved metabolic reconstructions suggest prospective functional synergies in biogeochemical processes. Collectively, these findings establish Patescibacteriota as a globally distributed, highly diverse lineage that not only contributes substantially to microbial biodiversity but also exerts critical influence on the functional dynamics of natural microbial consortia, providing new insights into its ecological significance and evolutionary adaptation.

## 2 Result and Discussion

### 2.1 RpS3 is a Robust Phylogenetic Marker for Patescibacteriota

To evaluate whether rpS3 can serve as a robust phylogenetic biomarker for characterizing Patescibacteriota communities in metagenomic datasets, three phylogenetic frameworks were constructed: an rpS3‐based tree, a 16S rRNA gene tree, and a genome tree derived from the concatenation of 120 single‐copy marker genes. Concordance among these trees was then assessed to determine the reliability of rpS3 as a phylogenetic marker.

From the 4645 genomes of Patescibacteria, 4314 rpS3 gene sequences and 2232 16S rRNA gene sequences were recovered (Figure S1a, Supporting Information). RpS3 sequences were significantly shorter than 16S rRNA genes (668 ± 35 vs 2364 ± 875 bp), a difference largely attributable to the unusually high frequency of insertions in Patescibacteriota 16S rRNA genes, where 918 of 2232 sequences contained at least one insertion, and some harbored up to seven (Figure S1b,c, Supporting Information).^[^ ^1^ ^]^ This atypical feature of Patescibacteriota 16S rRNA gene sequences reduces their recoverability from metagenomic data, whereas the shorter rpS3 sequences provided a recovery advantage, yielding nearly twice as many sequences from the same set of MAGs (4314 vs 2232).

Phylogenetic comparison further demonstrated the robustness of rpS3. The GTDB reference tree, constructed from 120 concatenated marker genes,^[^ ^2^ ^]^ resolved 24 Patescibacteriota classes, with >90% bootstrap support for 18 (Figure S2a, Supporting Information). The 16S rRNA gene tree recovered strong support (>90% bootstrap) for 14 class‐level clades, while the rpS3 tree provided comparable resolution, supporting 15 classes (Figure S2b, Supporting Information). Minor topological discrepancies were observed: the 16S rRNA tree subdivided class JAEDAM01 into two well‐supported clades, whereas the rpS3 phylogeny clustered two singletons (SOKK01 and GCA‐2792135) with classes JABMPQ01 and Patescibacteriia. Overall, both markers showed strong concordance with the GTDB taxonomy, with major classes forming well‐supported monophyletic groups.

Together, these results demonstrate that rpS3 functions as a robust phylogenetic marker for resolving Patescibacteriota diversity, consistent with recent applications in microbial ecology.^[^ ^25^, ^26^, ^27^ ^]^ To facilitate broader use, a curated rpS3 reference database comprising 57595 sequences was established, each taxonomically annotated based on GTDB‐classified source MAGs. This database enables standardized rpS3‐based community profiling and quantitative abundance estimation from metagenomes. In subsequent analyses, this strategy was integrated with MAG‐derived metabolic reconstructions, providing a complementary framework that maximizes diversity recovery while capturing genome‐resolved functional potential, thereby advancing the study of Patescibacteriota diversity and host‐linked metabolic interactions across ecosystems.

### 2.2 The RpS3 Biomarker Reveals the High Diversity of Patescibacteriota Across Diverse Ecosystems

**Figure 1:**
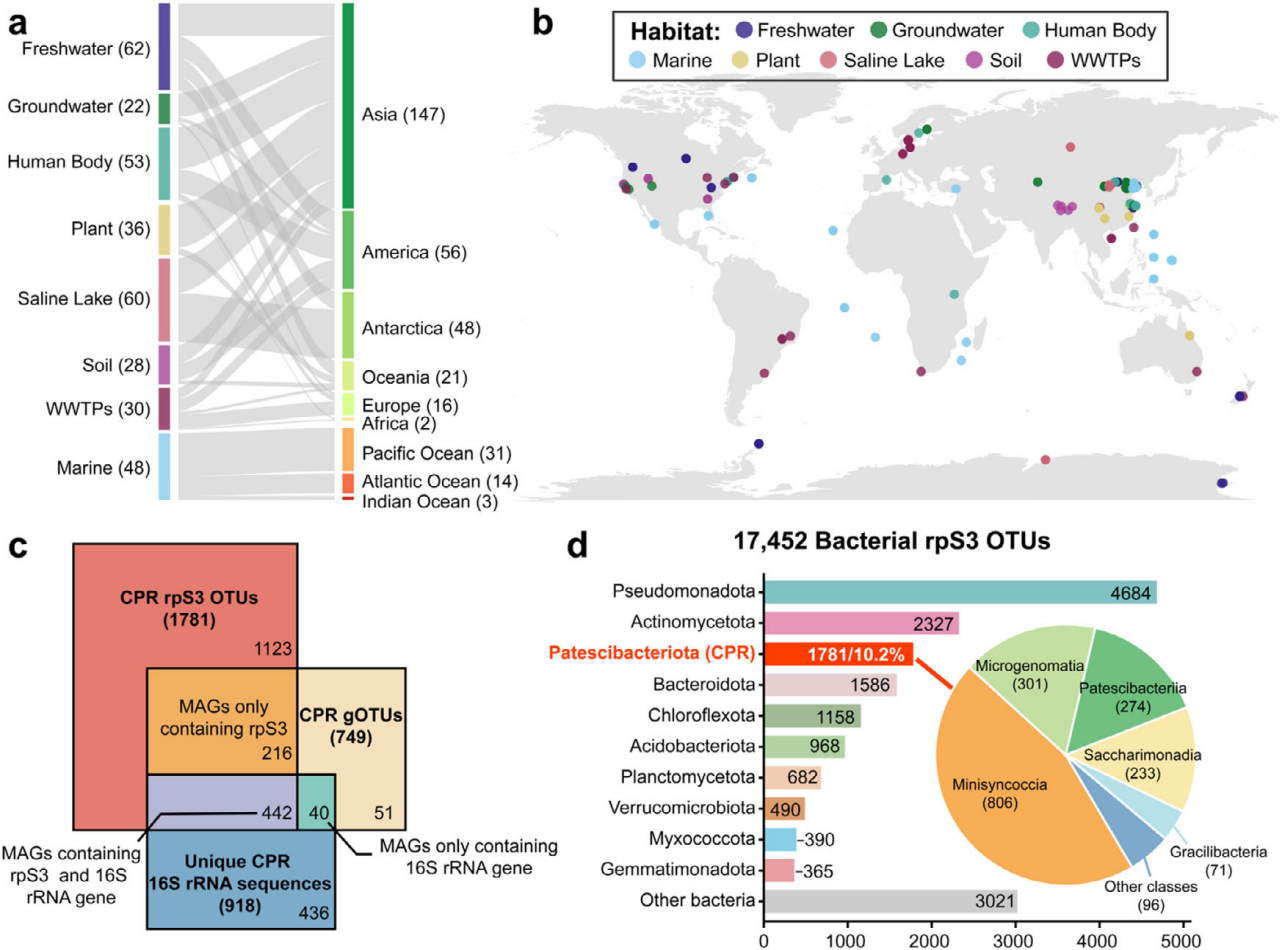
Overview of the global metagenomic sample datasets and evaluation of methods for Patescibacteriota diversity characterization. a) The number of samples categorized by habitat and continent. Bar heights indicate the number of samples per category, with habitat types color‐coded according to the following panel. b) Geographic distribution map of samples. Each point represents a site, colored by specific habitat classification. c) Comparison of Patescibacteriota diversity captured by three approaches: 16S rRNA gene sequences, rpS3 sequences, and MAGs. Species‐level thresholds were applied at 97% amino acid identity for rpS3 and 95% average nucleotide identity (ANI) for MAGs, whereas 16S rRNA genes were clustered at 100% identity, reflecting unique sequence variants rather than species‐level units. d) Taxonomic composition of bacterial rpS3 OTUs (bOTUs) at the phylum level (bar plot) and Patescibacteriota‐specific rpS3 OTUs (cprOTUs) at the class level (pie plot).

To evaluate the relative efficiency of rpS3‐based profiling compared with 16S rRNA genes‐ and MAG‐based approaches for characterizing the diversity of Patescibacteriota and bacteria in short‐read metagenomic datasets, we conducted a comprehensive analysis across multiple global habitats. A total of 339 metagenomic datasets (13.9 Tbp) from eight ecosystems—freshwater, groundwater, human body, marine, plant, saline lakes, soils, and wastewater treatment plants (WWTPs)—were analyzed (**Figure** **1**a,b; Table S1, Supporting Information). From these datasets, we recovered 27638 bacterial rpS3 sequences (including 2421 from Patescibacteriota), 8694 bacterial 16S rRNA sequences (including 991 from Patescibacteriota), and 9890 bacterial MAGs (including 906 from Patescibacteriota) after quality control.

To rigorously compare the performance of these approaches, we first assessed the intra‐species sequence identity of Patescibacteriota 16S rRNA genes and rpS3. Remarkably, 16S rRNA genes exhibited extreme divergence within a single species, with intra‐species identities ranging from 40.3% to 100% after removing the insertions within the 16S rRNA genes (Table S2, Supporting Information). A similar pattern was observed in Greengenes2,^[^ ^32^ ^]^ where intra‐species identities of Patescibacteriota 16S sequences varied from 44.3% to 100% (Table S2, Supporting Information). Importantly, this is not attributable to a few assembly artifacts but was observed in 41 of the 80 Patescibacteriota species examined, where intra‐species 16S rRNA similarity dropped below 85%, confirming that 16S rRNA genes in this lineage are unusually heterogeneous and lack a stable species‐level cutoff. By contrast, rpS3 sequences were far more conserved, typically ≥97% and in most cases >99% (Table S3, Supporting Information), consistent with earlier studies.^[^ ^25^, ^26^, ^27^ ^]^

We next benchmarked species‐level clustering thresholds: rpS3 amino acid sequences at 97% identity, MAGs at 95% ANI, while 16S rRNA gene sequences at 100% identity, the latter reflecting unique sequences rather than true species‐level units. Even under these stringent criteria, rpS3 retrieved substantially greater diversity, yielding 1781 Patescibacteriota rpS3 OTUs (cprOTUs) compared with 918 16S rRNA gene unique sequences and 749 genomic OTUs (gOTUs). After normalizing sequencing depth to 50 Gb, rpS3 consistently detected the largest number of Patescibacteriota sequences across all eight habitats (Figure S3, Supporting Information). Collectively, these results demonstrate that rpS3 provides superior resolution of Patescibacteriota diversity from short‐read metagenomic datasets compared with both MAG‐ and 16S‐based approaches.

In total, 17452 bacterial rpS3 OTUs were obtained at the species level, of which 1781 (10.2%) belonged to Patescibacteriota. This lineage ranked as the third most diverse bacterial phylum after Pseudomonadota (24.3%) and Actinomycetota (12.5%) (Figure 1c). Phylogenetic analysis revealed that the 1781 Patescibacteriota rpS3 OTUs encompassed 15 of 24 recognized classes, with two OTUs remaining unclassified at the class level. Five classes—Minisyncoccia, Microgenomatia, Patescibacteriia, Saccharimonadia, and Gracilibacteria—dominated the diversity, accounting for 94.6% of all Patescibacteriota OTUs (Figure 1c; Table S2, Supporting Information). Together, these findings establish Patescibacteriota as one of the most diverse bacterial phyla, with its diversity spanning multiple evolutionary lineages but strongly structured around several dominant clades.

### 2.3 Global Distribution of Patescibacteriota Exhibits Habitat Heterogeneity

**Figure 2:**
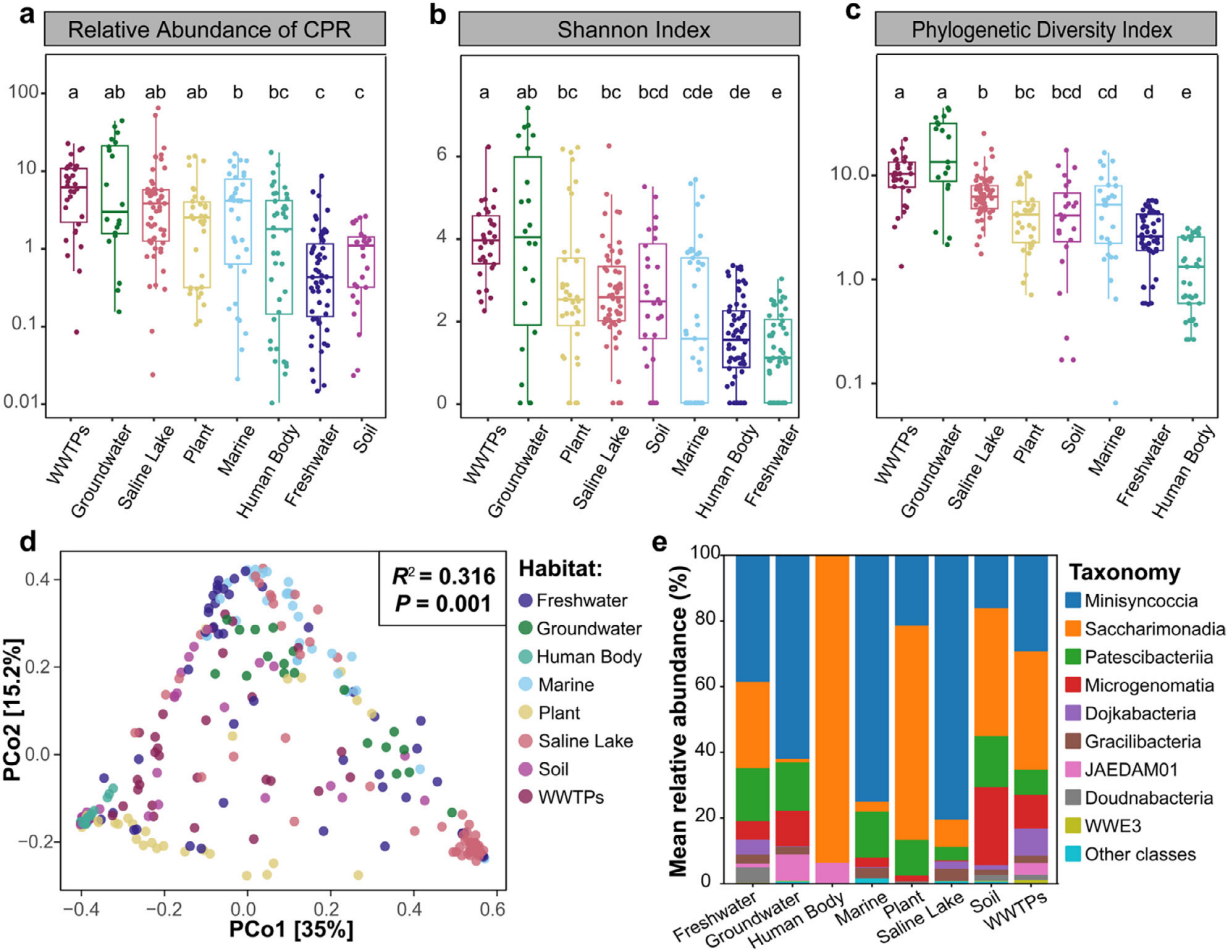
Habitat‐specific distribution patterns of Patescibacteriota. Box plots comparing Patescibacteriota communities across habitats for a) relative abundance (log‐transformed), b) Shannon diversity index, and c) Faith's phylogenetic diversity (PD). Boxes represent interquartile ranges (IQR) with median lines; whiskers extend to 1.5×IQR. Lowercase letters indicate statistically distinct groups (Games‐Howell post‐hoc test, *P* < 0.05). d) Principal coordinates analysis (PCoA) of Patescibacteriota community composition based on weighted UniFrac distances. Axes indicate the percentage of variance explained. e) Class‐level taxonomic composition of Patescibacteriota communities across habitats.

The global distribution of Patescibacteriota was examined across 339 metagenomic samples spanning eight ecosystems. rpS3‐based OTUs at the species level revealed Patescibacteriota presence in 296 samples (87.3%), typically comprising 0.1‐10% of the total bacterial community. The Games‐Howell test indicated significant differences in relative abundance among habitats, with the highest levels detected in groundwater (10.96 ± 13.49%) and WWTPs (7.37 ± 5.94%) (**Figure** **2**a), followed by saline lakes, plant, marine, human body, freshwater, and soil. Particularly striking was the enrichment observed in two BTEX‐contaminated groundwater samples (GW_20 and GW_21 in Table S1 (Supporting Information), benzene: 1,340 mg L^−1^; toluene: 1,000 mg L^−1^; ethylbenzene: 133 mg L^−1^; and xylene: 1,420 mg L^−1^),^[^ ^33^ ^]^ where Patescibacteriota accounted for 31.3‐37.4% of the total bacterial community. Similarly, a previously reported agriculturally impacted groundwater with elevated nitrate concentrations (195 mg L^−1^) contained ≈40% Patescibacteriota.^[^ ^26^ ^]^ In addition, Patescibacteriota reached 65% of the total bacterial community in a saline lake (SL_09 in Table S1, Supporting Information) characterized by active sulfate reduction.^[^ ^34^ ^]^ Collectively, these findings suggest potential ecological roles for Patescibacteriota in nitrogen and sulfur cycling (see Sections 2.5 and 2.6) and possibly in pollutant degradation, mediated through interactions with their microbial hosts.

Then we compared the alpha‐diversity of Patescibacteriota communities across eight distinct habitats. The phylogenetic diversity (PD) and Shannon indices of the Patescibacteriota communities in WWTPs (PD index: 10.48 ± 4.82; Shannon index: 3.57 ± 0.82) and groundwater (PD index: 17.46 ± 15.03; Shannon index: 3.46 ± 2.20) were significantly higher than those in other habitats, followed by saline lake (PD index: 6.58 ± 4.13; Shannon index: 2.39 ± 1.11), plant (PD index: 4.10 ± 3.09; Shannon index: 2.53 ± 1.63), soil (PD index: 4.44 ± 4.45; Shannon index: 2.53 ± 1.63), marine (PD index: 3.43 ± 4.41; Shannon index: 1.64 ± 1.67), freshwater (PD index: 2.39 ± 1.82; Shannon index: 1.39 ± 0.95) and human body (PD index: 1.01 ± 1.10; Shannon index: 1.08 ± 0.90). Notably, groundwater and WWTPs, which are research hotspots,^[^ ^1^, ^24^, ^26^, ^35^ ^]^ exhibited both the highest abundance and diversity of Patescibacteriota (Figure 2b,c). It is worth noting that although groundwater represents one of the habitats with the highest Patescibacteriota diversity, the corresponding rarefaction curve has not yet reached saturation (Figure S4, Supporting Information). Additionally, high abundance and diversity of Patescibacteriota were also observed in plants and saline lakes, suggesting these habitats have the potential to become new hotspots for Patescibacteriota research.

The measurement of beta‐diversity revealed significant differences in the Patescibacteriota community across habitats. Principal coordinate analysis based on weighted UniFrac distance revealed that the Patescibacteriota communities of different habitats formed eight distinct clusters (PERMANOVA, *R^2^* = 0.316, *P* = 0.001) (Figure 2d). Across the 8 habitats, Patescibacteriota communities were mainly dominated by four classes (83.16‐99.32% total abundance): Minisyncoccia, Saccharimonadia, Patescibacteriia, and Microgenomatia. The habitat preferences were detectable at the class level. Saccharimonadia was most abundant in the human body (93.64 ± 12.57%), plant (65.19 ± 35.07%), and soil (38.94 ± 41.86%), while Minisyncoccia dominated in groundwater (61.94 ± 22.78%), saline lake (80.40 ± 19.38%), and marine (75.02 ± 29.97%) (Figure 2e). LEfSe analysis (LDA > 4.0, *P* < 0.05) identified 24 Patescibacteriota families with significant abundance differences, including human‐specific obligate symbionts such as Nanosynbacteraceae and Nanosyncoccaceae from human oral environments,^[^ ^36^ ^]^ as well as the family UBA918, which was widely distributed (detection rate: 0–63.3%, mean abundance: 0–0.3%) but specifically enriched in saline lake (detection rate: 78.3%, mean abundance: 3.6%) (Figure S5, Supporting Information). These results suggest habitat heterogeneity in the community structure of Patescibacteriota. The mechanisms driving habitat‐specific differentiation of Patescibacteriota communities, including nutrient availability, host dependence, and physicochemical constraints, remain to be elucidated in future studies.

### 2.4 The Co‐Occurrence Network Revealed the Host Dependence and Habitat Specificity of Patescibacteriota Occurrence

**Figure 3:**
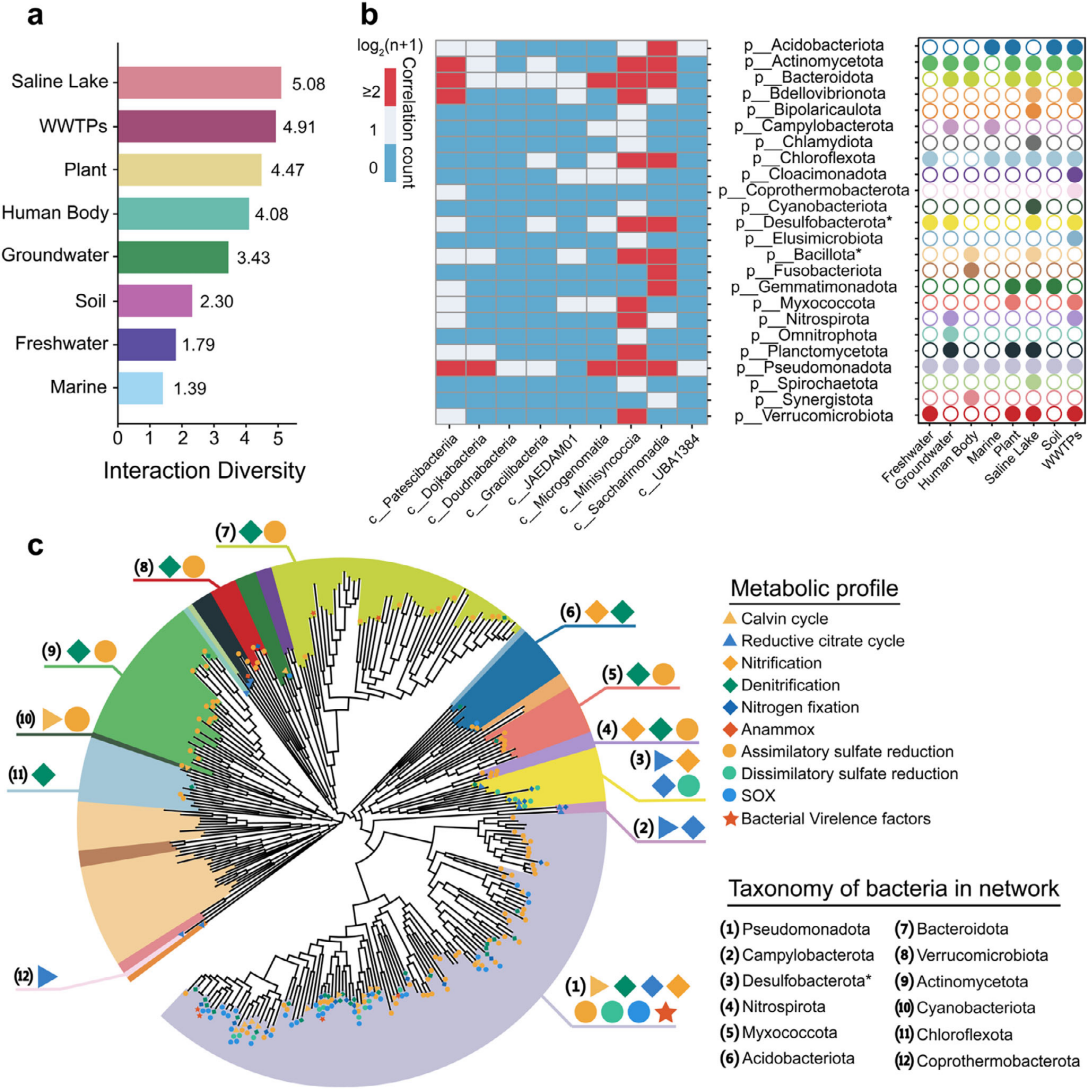
Interaction networks between Patescibacteriota and their putative bacterial hosts. a) Habitat‐specific interaction diversity in Patescibacteriota‐host bipartite networks. Bars represent Shannon entropy values of edge weights (indicating interaction strength heterogeneity) across habitats. Colors correspond to habitat classifications. b) Heatmap illustrating the network associations between Patescibacteriota at the class level and other bacteria at the phylum level. The color represents the number of observed associations (log_2_(n+1)) between cprOTUs and bOTUs. The bubble chart shows the habitat distribution of Patescibacteriota‐bacteria network associations. c) Phylogenetic reconstruction and metabolic profiling of hosting bOTUs matched to reference genomes, revealing an indirect role of Patescibacteriota in microbiome via interaction with hosts. The Maximum likelihood tree was constructed using FastTree with the ML model WAG. Taxon groups are represented by colored branches, consistent with the phylum‐level color scheme shown on the right side of (b). Metabolic functions are indicated by symbols: triangles (carbon metabolism), diamonds (nitrogen metabolism), circles (sulfur metabolism), and stars (bacterial virulence factors).

To predict potential symbiotic relationships between Patescibacteriota and other bacteria, we employed a self‐designed bootstrap sampling‐based network analysis using Spearman correlation coefficients to infer network associations across eight habitats.^[^ ^37^ ^]^ This analysis identified a total of 494 associations between 174 cprOTUs and 318 non‐Patescibacteriota bacterial rpS3 OTUs (bOTUs) across investigated habitats (Figure S6; Table S5, Supporting Information). The 174 cprOTUs were distributed across nine distinct Patescibacteriota classes, with Minisyncoccia, Saccharimonadia, and Patescibacteriia exhibiting the highest frequency of co‐occurrence relationships. These three classes collectively accounted for 88.9% (439/494) of the total observed co‐occurrences, suggesting their potential ecological significance within microbial community networks. Furthermore, the 318 bOTUs associated with cprOTUs were distributed across 24 distinct bacterial phyla. Among these, Pseudomonadota, Actinomycetota, and Bacteroidota demonstrated the highest frequency of network associations with Patescibacteriota taxa (**Figure** **3**b). This pattern suggests potential preferential interactions between Patescibacteriota and these dominant bacterial phyla within the microbial community structure.

The potential interaction relationships revealed by our network analyses were consistent with previously reported pure culture‐based or network‐based studies. For instance, the network association between cprOTU960 and bOTU8989 mirrors the experimentally validated symbiotic relationship between Patescibacteriota strain Southlakia epibionticum (*Se*) ML1 and its host Actinomyces israelii (*Ai*) F0345 at the OTU level.^[^ ^28^ ^]^ Specifically, cprOTU960 shared 100% rpS3 sequence identity with Patescibacteriota strain *Se* ML1, while bOTU8989, which co‐occurred with cprOTU960, exhibited 97.9% rpS3 sequence identity with the host strain *Ai* F0345. Moreover, the intensive associations observed between Saccharimonadia and Actinomycetota, as well as between Saccharimonadia and Bacteroidota, were also consistent with previous reports.^[^ ^38^ ^]^ Collectively, these results suggest that our network analysis largely captures the ecological associations between Patescibacteriota and their potential hosts.

Host‐Patescibacteriota network analyses revealed that greater interaction diversity between Patescibacteriota and their hosts tended to lead to higher α‐diversity of Patescibacteriota across habitats. Using the Shannon index to quantify host‐Patescibacteriota interaction diversity, we identified the following habitat‐specific gradient: saline lakes > WWTPs > plants > human body > groundwater > soil > freshwater > marine (Figure 3a). Linear regression further confirmed the significant positive relationship between interaction diversity and α‐diversity of Patescibacteriota (Figure S7, Supporting Information). These findings suggest that the occurrence and diversification of Patescibacteriota are strongly dependent on their interactions with hosts, consistent with their limited metabolic capacity and predominantly epibiotic lifestyle reliant on host‐derived resources.^[^ ^4^ ^]^

Furthermore, the host‐Patescibacteriota network exhibited apparent habitat specificity. Across all datasets, 24 bacterial phyla were identified as potential hosts, with 7 phyla showing associations in over half of the habitats (4 of 8); notably, these hosts consistently ranked among the top 10 most abundant phyla (Table S6, Supporting Information). We define these as broadly distributed potential host‐Patescibacteriota interactions. By contrast, 15 of the 24 phyla displayed associations in only one or two habitats, despite being present in more than two habitats and often reaching relatively high abundances (2–5%, Table S6, Supporting Information). This indicates that mere coexistence does not necessarily lead to host‐Patescibacteriota interactions. Instead, habitat‐specific biogeochemical processes likely constitute the critical conditions enabling such associations. For instance, Nitrospirota showed strong associations with Patescibacteriota in WWTPs environments, but such interactions were absent in freshwater habitats, despite Nitrospirota being significantly less abundant in the former than in the latter (0.6% vs 1.8%, Table S6, Supporting Information). This may be attributable to the more active nitrogen cycling processes characteristic of WWTPs. Thus, host‐Patescibacteriota interactions are inherently habitat‐dependent.

It is noteworthy that hosts potentially interacting with Patescibacteriota generally participate in key CNS cycling processes. To evaluate whether a host phylum was involved in a specific process, we required that at least three MAGs within that phylum each contained ≥70% of the functional genes associated with the corresponding pathway, and only under this criterion was the phylum designated as contributing to biogeochemical cycling. Based on this standard, 5 host phyla showed potential for CO_2_ fixation, although none encoded a relatively complete methane metabolism or methanogenesis pathway (Figure 3c); 10 phyla were implicated in nitrogen cycling, including 8 with potential for denitrification, 3 for nitrification and 2 for nitrogen fixation (Figure 3c); and 8 phyla were involved in sulfur cycling, with 8 harboring genes for sulfate metabolism and one for thiosulfate metabolism (Figure 3c). Additionally, one host phyla possessed genes potentially associated with virulence factors (Figure 3c; Table S7, Supporting Information). Considering that CPR may influence host growth rates and population size,^[^ ^39^, ^40^ ^]^ we infer that CPR could indirectly shape biogeochemical cycling through their interactions with metabolically active hosts.

### 2.5 Nitrogen‐Sulfur Co‐Metabolism Between Denitrifying Patescibacteriota and Desulfobacterota

Our network analysis revealed significant associations between Patescibacteriota and SRB Desulfobacterota (Table S5, Supporting Information). The network identified 16 pairs of co‐occurrence relationships between 16 cprOTUs (belonging to 14 genera) and 10 bOTUs of Desulfobacterota (Table S5, Supporting Information). Notably, Patescibacteriota genera *OLB19* and *C7867‐001* each contained two cprOTUs associated with Desulfobacterota. Interestingly, 7 of the 16 cprOTUs hold the potential to encode the nitrite reductase (*nirK*) gene, particularly in genera *OLB19* and *C7867‐001* (Figure S8, Supporting Information). Moreover, *nirK* was not restricted to the two cprOTUs most strongly linked to *Desulfobacterota* in the network; rather, it was broadly distributed across multiple genomes within OLB19 (n = 5) and C7867‐001 (n = 11) (Figure S8, Supporting Information). This suggests that potential interactions with SRB may involve multiple members of these two Patescibacteriota genera, although such interactions are unlikely to apply universally to all species within these lineages. Crystal structure prediction showed that Patescibacteriota NirK proteins have high homology with classical NirK (*Hyphomicrobium denitrificans*, PDB ID: 2DV6, TM‐scores: 0.881 and 0.907), containing key catalytic active sites D‐H (Figure S9, Supporting Information),^[^ ^41^ ^]^ suggesting its activity in catalyzing nitrite (NO_2_^−^) conversion to NO. Thus, the network indicates potential symbiotic relationships between Desulfobacterota and denitrifying Patescibacteriota with denitrification potential, enabling coupled sulfur–nitrogen metabolism within microbial consortia.

**Figure 4:**
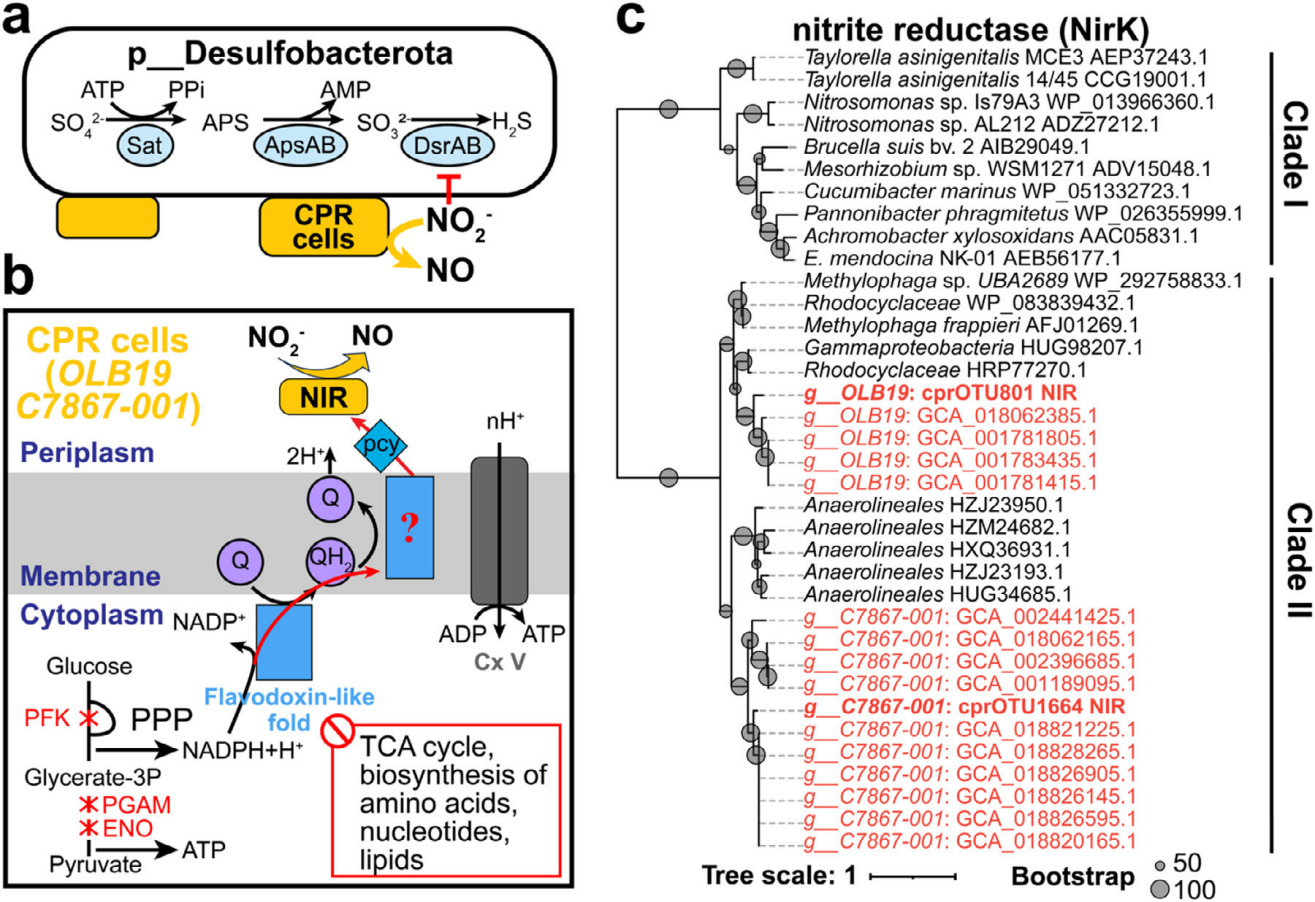
Evolutionary and functional evidence for Patescibacteriota‐encoded NirK in mitigating nitrite stress for Desulfobacterota (SRB) hosts. a) Model of metabolic synergies between Patescibacteriota and Desulfobacterota, in which Patescibacteriota cells (yellow) may assist Desulfobacterota in resisting nitrite stress. b) Predicted metabolic capacities of Patescibacteriota (genera *OLB19* and *C7867‐001*) associated with Desulfobacterota, highlighting the presence of NirK and other functional genes potentially involved. c) Maximum likelihood tree from amino acid alignments of Patescibacteriota NirK and reference microbial NirK sequences. The branches highlighted in red correspond to Patescibacteriota genomes, while the branches in bold red represent NirK of cprOTUs. The phylogenetic tree was constructed using IQ‐TREE with the LG+I+R7 model. Abbreviations: TCA cycle, Tricarboxylic acid cycle; PPP, Pentose phosphate pathway; Sat, sulfate adenylyltransferase; Aps, adenylylsulfate reductase; Dsr, dissimilatory sulfite reductase; NIR, nitrite reductase; pcy, plastocyanin; Cx V, Complex V (ATP synthase); PFK, phosphofructokinase; PGAM, 2,3‐bisphosphoglycerate‐dependent phosphoglycerate mutase; ENO, enolase.

Patescibacteriota with NO_2_^−^ reduction capability may enhance the ecological advantage of host Desulfobacterota in high NO_2_^−^ environments (**Figure** **4**). It has been documented that 5–50 ppm of nitrite can inhibit SRB activity in oil, gas, and wastewater reservoirs.^[^ ^42^ ^]^ Cultivation experiments demonstrate that nitrite significantly downregulates genes related to sulfate reduction, energy metabolism, and electron transfer in SRB, thereby inhibiting SRB activity.^[^ ^43^ ^]^ Our study found that network associations between Desulfobacterota and denitrifying cprOTUs occur more frequently in ecosystems such as WWTPs and groundwater (7/7, Table S5, Supporting Information), which are high‐risk habitats for NO_2_^−^ pollution. Consequently, denitrifying CPR may enhance the adaptability of host Desulfobacterota to NO_2_^−^‐polluted environments. Consistently, metatranscriptomic analyses detected co‐transcription of the denitrifying Patescibacteriota *nirK* gene together with the SRB functional genes *dsrA* and *dsrB* in these habitats (Table S8, Supporting Information), further supporting potential ecological interactions between these two groups. Taken together, these findings suggest that denitrifying Patescibacteriota may enhance the adaptability of Desulfobacterota to nitrite‐rich environments, although definitive validation will require future strain‐based cultivation experiments.

Comparative genomics indicated that SRB‐associated Patescibacteriota may possess unique electron transport chains (ETC) that differ from those of canonical prokaryotes (Figure 4). All SRB‐associated Patescibacteriota MAGs exclusively encode NADPH‐quinone reductase and ubiquinone synthesis‐related genes, indicating their ability to transfer electrons from NADPH to ubiquinone. Additionally, these MAGs encode a protein with a single cupredoxin domain, annotated as plastocyanin (pcy), suggesting that pcy proteins function as electron carriers transferring electrons to NirK proteins. However, the SRB‐associated Patescibacteriota MAGs from the genera *OLB19* and *C7867‐001* universally lack genes encoding complex III, which appears to be replaced by an as‐yet unidentified non‐classical protein that mediates electron transfer from ubiquinone to pcy. While genome incompleteness and bin contamination may distort gene presence/absence, these features are unlikely to be artifacts, as the absence of complex III is a widespread characteristic across Patescibacteriota genomes and not limited to SRB‐associated MAGs. Nevertheless, it should be noted that even when a gene is consistently observed across multiple MAGs from the same OTU, strain‐level heterogeneity means that not every genome within that OTU necessarily encodes the gene.

It is worth noting that the presence of *nirK* genes in Patescibacteriota does not necessarily indicate metabolic interactions with Desulfobacterota. Patescibacteriota exhibit some host specificity,^[^ ^40^ ^]^ possibly determined by factors such as the specificity of cell envelope‐associated proteins.^[^ ^28^ ^]^ Through systematic analysis of 4645 Patescibacteriota genomes, we found that over 50 Patescibacteriota lineages, comprising more than 140 Patescibacteriota MAGs, encode the *nirK* gene, indicating that the presence of *nirK* in Patescibacteriota is a relatively common phenomenon, far exceeding the seven relationships explained by the network. These results suggest that the presence of the *nirK* gene is not a sufficient condition for Patescibacteriota‐Desulfobacterota interactions. Furthermore, cprOTUs associated with Desulfobacterota that lack the *nirK* gene may indicate other unknown metabolic interactions between Desulfobacterota and Patescibacteriota, or could be due to incomplete MAG assembly resulting in *nirK* gene loss, or false positives arising from mathematical correlations in the network. Our results indicate that symbiosis with denitrifying Patescibacteriota may provide ecological benefits to Desulfobacterota and facilitate S‐N coupling, although this remains to be validated by strain‐level cultivation studies.

### 2.6 Nitrogen‐Nitrogen Co‐Metabolism Between Denitrifying Patescibacteriota and *Nitrospira*

**Figure 5:**
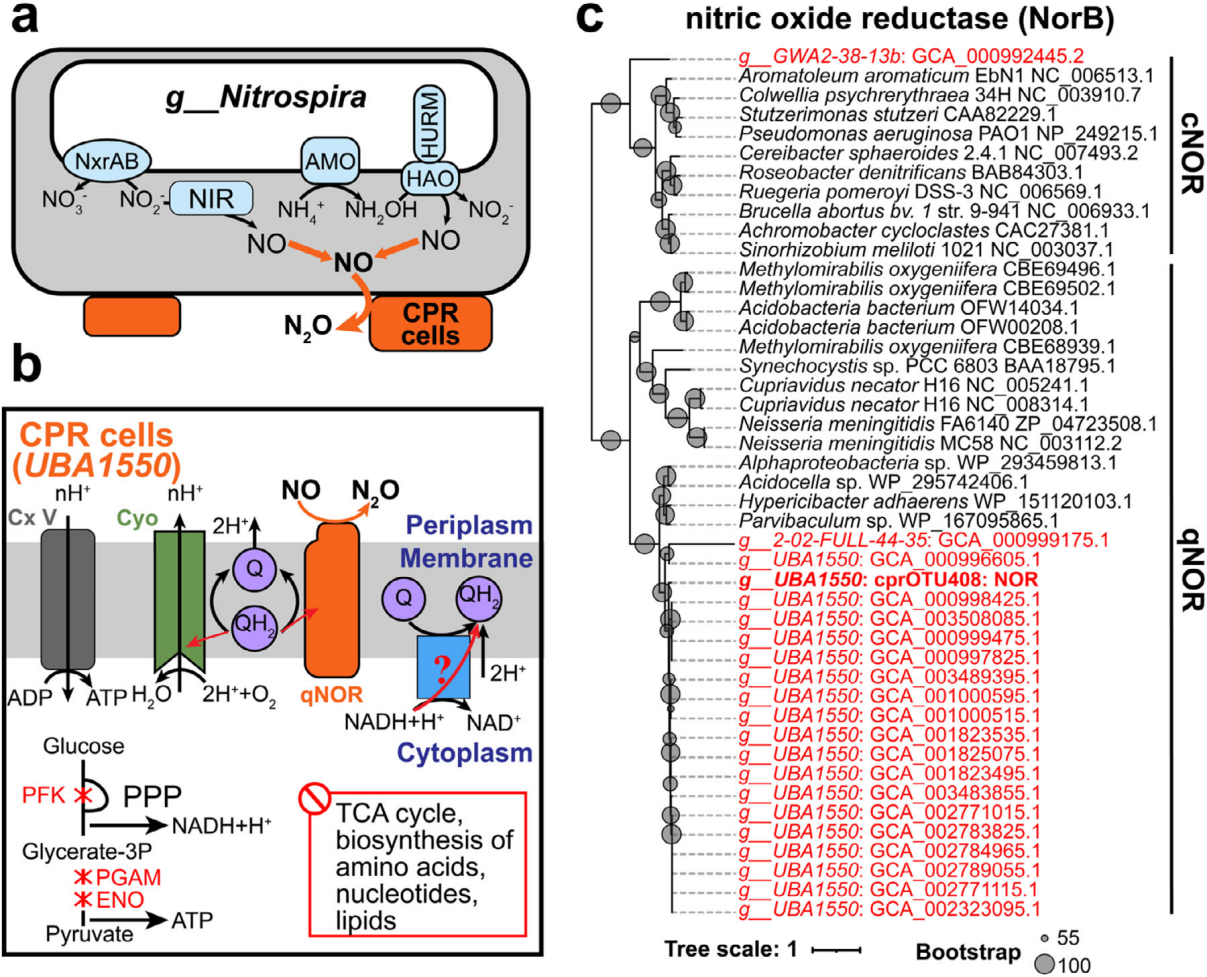
Evolutionary and functional evidence for Patescibacteriota‐encoded NorB in mitigating NO stress for hosts. a) Model of metabolic cascade between Patescibacteriota and CMX, in which Patescibacteriota cells (orange) may mitigate NO toxicity for CMX. b) Predicted metabolic capacities of Patescibacteriota (genus *UBA1550*) associated with *Nitrospira*. c) Maximum likelihood tree from amino acid alignments of Patescibacteriota NorB and reference microbial NorB sequences. The branches highlighted in red correspond to Patescibacteriota genomes, while the branch in bold red represents NorB of cprOTU408. The phylogenetic tree of NorB was constructed using IQ‐TREE with the LG+F+G4 model. Abbreviations: TCA cycle, Tricarboxylic acid cycle; PPP, Pentose phosphate pathway; Nxr, nitrite oxidoreductase; NIR, nitrite reductase; AMO, ammonia monooxygenase; HAO, hydroxylamine dehydrogenase; HURM, hydroxylamine‐ubiquinone reaction module; Cx V, Complex V (ATP synthase); Cyo, Cytochrome o ubiquinol oxidase; qNOR, quinol‐dependent nitric oxide reductases; PFK, phosphofructokinase; PGAM, 2,3‐bisphosphoglycerate‐dependent phosphoglycerate mutase; ENO, enolase.

The network analysis also revealed potential N‐N metabolic interactions between Patescibacteriota with partial denitrification capabilities and their potential host *Nitrospira*. Significant network associations were observed between six cprOTUs and three bOTUs from the genus *Nitrospira*, especially two cprOTUs belonging to the genus *UBA1550* and one *Nitrospira* bOTU (Table S5, Supporting Information). The representative MAG of cprOTU408, as well as 18 additional genomes belonging to the same genus, consistently harbored the *norB* gene (**Figure** **5**b), which encodes a protein that catalyzes the reduction of NO to N_2_O. This enrichment of *norB* appears to be specific to *UBA1550*, as other Patescibacteriota genera contained at most a single *norB* gene and showed no comparable enrichment. The potential host *Nitrospira*, traditionally recognized as typical nitrifying microbes, can drive the oxidation of NO_2_^−^ to nitrate (NO_3_^−^).^[^ ^44^ ^]^ Genomic studies have found that almost all *Nitrospira* contain the *nirK* gene,^[^ ^44^ ^]^ which is speculated to form NO from NO_2_^−^, potentially providing electron acceptors for denitrifying Patescibacteriota containing the *norB* gene. Therefore, this study suggests that these denitrifying Patescibacteriota within the genus *UBA1550* containing the *norB* gene may have N‐N metabolic interaction potential with *Nitrospira*, jointly driving the nitrogen biogeochemical cycle (Figure 5a).

*Nitrospira* may gain an ecological advantage through symbiosis with denitrifying Patescibacteriota containing the *norB* gene, which uses NO as an electron acceptor. The genus *Nitrospira* includes not only canonical nitrite‐oxidizing bacteria (NOB) but also the recently discovered complete ammonia‐oxidizing microorganisms (comammox, CMX). CMX, belonging to lineage II of the genus *Nitrospira*, is the latest discovered group of nitrifying microorganisms capable of oxidizing ammonia to nitrate within a single cell.^[^ ^45^, ^46^ ^]^ Previous studies have shown that in classical 16S rRNA gene phylogenetic trees, CMX and traditional NOB do not form significantly different subclades.^[^ ^44^, ^45^, ^46^, ^47^ ^]^ However, in genome‐based phylogenetic trees, CMX and NOB within lineage II form distinct, robustly supported subclades. Based on 42 genomes collected from the three bOTUs of *Nitrospira* in the network, our genomic phylogenetic analysis revealed that among these three bOTUs, one (bOTU7758) belongs to *Nitrospira* Lineage I NOB, while the other two (bOTU4345 and bOTU13121) belong to *Nitrospira* Lineage II clade A CMX (Figure S10, Supporting Information). Notably, genomes from both lineage I NOB and CMX clade A contain the *nirK* gene, responsible for converting NO_2_^−^ to NO, and CMX clade A can also generate NO via hydroxylamine oxidation catalyzed by *hao*. During nitrifier‐denitrification or hydroxylamine oxidation, NO can accumulate intracellularly if not rapidly detoxified or reduced to N_2_O, potentially leading to cellular damage.^[^ ^48^, ^49^ ^]^ Moreover, NO can be used as an electron acceptor by Patescibacteriota containing the *norB* gene that coexist with CMX, potentially enabling N‐N metabolic interactions between Patescibacteriota and their hosts, jointly driving the nitrogen biogeochemical cycle. Supporting this possibility, metatranscriptomic datasets revealed co‐expression of Patescibacteriota *norB* with CMX functional genes *amoA*, *amoB*, and *hao* (Table S8, Supporting Information). Collectively, these findings suggest that denitrifying Patescibacteriota may contribute to host resilience in NO‐rich niches, though definitive validation will require strain‐based co‐culture experiments.

Comparative genomic analysis revealed that *UBA1550* Patescibacteriota symbiotic with *Nitrospira* possess a unique ETC distinct from classical denitrifying microorganisms. The NorB encoded by *UBA1550* is a quinone‐dependent nitric oxide reductase (qNOR) that can accept electrons from quinones to convert NO to N_2_O. Crystal structure prediction showed that the NorB protein encoded by *UBA1550* has high homology (TM‐score: 0.955) with known NorB proteins (*Alcaligenes xylosoxidans*, PDB ID: 8BGW) (Figure S11, Supporting Information). Sequence analysis indicated that the NorB encoded by *UBA1550* contains conserved active site residues H‐E.^[^ ^50^ ^]^ Although lacking the classical ETC like most Patescibacteriota, the denitrifying Patescibacteriota symbiotic with *Nitrospira* encode qNOR (requiring quinone electrons) and cytochrome o quinol oxidase (CyoABCD), suggesting the possible existence of an unknown protein or mechanism maintaining electron transfer to achieve electron transfer from NADH to quinone (Figure 5).

## 3 Conclusion

This study establishes rpS3 as a robust phylogenetic marker that substantially improves recovery of Patescibacteriota diversity from short‐read metagenomic datasets, while complementing MAG‐based analyses to provide a more comprehensive view of both taxonomic structure and functional potential. By integrating 13.9 Tb of metagenomic datasets spanning eight ecosystems with 4645 Patescibacteriota MAGs, we demonstrate that this phylum represents one of the most phylogenetically diverse bacterial lineages, characterized by pronounced community heterogeneity and strong host dependencies across habitats. Network analyses further reveal habitat‐specific symbiotic associations, highlighting their ecological contributions through nitrogen‐sulfur co‐metabolism with Desulfobacterota and nitrogen‐nitrogen co‐metabolism with *Nitrospira* clade A comammox (Figure S12, Supporting Information). These findings not only expand current understanding of the ecological roles of Patescibacteriota but also underscore their importance as metabolic partners shaping biogeochemical cycling in diverse environments.

## 4 Experimental Section

### Ribosomal Protein S3 Database Construction

For the rpS3 taxonomy assignment, an rpS3 protein database was built based on GTDB R207.^[^ ^2^ ^]^ The GTDB was a microbial genome database that provides standardized taxonomic annotations based on genomic phylogeny. GTDB R207 organizes 317542 prokaryotic genomes into 65703 species clusters, within which Patescibacteriota comprises 2485 species groups encompassing 4645 MAGs. To comprehensively characterize bacterial taxonomy, rpS3 sequences were extracted from representative genomes of all species groups (n = 65703) in GTDB R207. Each rpS3 sequence was assigned a taxonomic classification based on the GTDB classification of its corresponding genome. Specifically, the representative genomes of all species groups from GTDB were downloaded. Then, open reading frames (ORFs) were predicted for each representative genome using Prodigal (v2.6.3) and the rpS3 protein was identified through alignment against the KEGG (v94.0) database using DIAMOND (v2.1.9) with at least 50% sequence coverage and 30% identity.^[^ ^51^, ^52^, ^53^ ^]^ Finally, an rpS3 database was established, consisting of 57595 rpS3 sequences with definitive species classifications.

### Comparative Phylogenetic Analysis of Patescibacteriota Based on GTDB R207

For comparative phylogenetic analysis, all Patescibacteriota genomes (n = 4645) were downloaded from NCBI using the genome accessions provided in the metadata of the GTDB R207 genome. Subsequently, both rpS3 protein sequences and 16S rRNA gene sequences were extracted from all Patescibacteriota genomes. ORFs of rpS3 protein sequences were predicted using Prodigal and identified by searching against the KEGG database using DIAMOND with 50% query coverage and 50% subject coverage. The Patescibacteriota 16S rRNA gene sequences were identified based on a hidden Markov model (HMM) search with manually curated structural alignment of the 16S rRNA by using the script provided by Banfield.^[^ ^1^ ^]^ After removing insertions, SeqKit (v2.8.2) was used to analyze the length distribution of both rpS3 protein sequences and 16S rRNA gene sequences.^[^ ^54^ ^]^ Only 16S rRNA gene sequences exceeding 1000 bp and rpS3 protein sequences longer than 200 amino acids were retained for the construction of phylogenetic trees. The script also identified the insertions in Patescibacteriota 16S rRNA gene sequences and provided both the length and number of these insertions.

The multiple sequence alignments of the concatenated phylogenetic marker genes were generated with GTDB‐Tk (v2.1.1) using the “identify” and “align” modules.^[^ ^55^ ^]^ Additionally, multiple sequence alignments of rpS3 and 16S rRNA gene were generated using MAFFT (v7.407) and trimmed with Gblocks (v0.91b).^[^ ^56^, ^57^ ^]^ Phylogenetic trees of the concatenated marker genes, 16S rRNA gene, and rpS3 were then constructed using FastTree (v2.1.11) under the WAG model.^[^ ^58^ ^]^ All these trees were visualized and refined on the iTOL web server.^[^ ^59^ ^]^

### Publicly Sample collection

Based on the isolation source information of Patescibacteriota MAGs in GTDB R207, the primary habitats of Patescibacteriota MAGs were classified into several categories: freshwater, groundwater, human body, marine, plant, saline lake, soil, WWTPs, and other sample types (including enrichment cultures, animal‐associated samples, etc.). To systematically investigate the distribution patterns of Patescibacteriota across diverse habitats, 318 publicly available samples were collected from NCBI (National Center for Biotechnology Information), ENA (European Nucleotide Archive), CNCB (China National Center for Bioinformation), and 21 in‐house samples, covering the following major habitats: freshwater (n = 62), groundwater (n = 22), human body (n = 53), marine (n = 48), saline lake (n = 60), plant (n = 36), soil (n = 28), and WWTPs (n = 30). Specifically, keyword‐based searches were conducted in NCBI, ENA, and TerrestrialMetagenomeDB.^[^ ^60^ ^]^ To ensure sample representativeness, considerable efforts were made to incorporate samples with diverse geographic distributions across different continents.

### Sampling, DNA Extraction, and Sequencing

In‐house samples used in this study were sourced from multiple research projects, both published and unpublished (see details in Table S1, Supporting Information). In brief, soil samples were collected and transported to the laboratory on dry ice, then stored at −80 °C until DNA extraction. For water samples, on‐site filtration was performed by passing 1–2 L of water through 0.2 µm polyethersulfone (PES) membrane filters (PALL Corporation). The membrane filters were immediately preserved on dry ice during transportation and subsequently stored at −80 °C upon arrival at the laboratory. Total DNA was extracted from both soil samples (0.5 g each) and membrane filters using the FastDNA SPIN Kit for Soil (MP Biomedicals, Solon, OH, USA) according to the manufacturer's instructions. All samples were sequenced as 150 bp paired‐end reads on the Illumina NovaSeq 6000 platform (Guangdong Magigene Biotechnology Co., Ltd., China).

### Metagenomic Assembly and Genome Binning

The NCBI SRA Toolkit (v3.0.5) was used to retrieve and process metagenomic data from NCBI and ENA. Adapter sequences and low‐quality bases (Q < 30) were trimmed using Trimmomatic (v0.39),^[^ ^61^ ^]^ retaining only reads with lengths of 50 bp or more. For human‐related metagenomic data, host genome contamination was removed by mapping raw reads to the human reference genome GRCh37 (hg19) using Bowtie2 (v2.4.1), excluding any reads aligning to the host genome.^[^ ^62^ ^]^ Cleaned reads were then assembled using Megahit (v1.1.3) with default parameters.^[^ ^63^ ^]^ Bacterial rpS3 sequences were identified by searching ORFs (predicted from contigs using Prodigal) against the ribosomal protein database and KEGG database using DIAMOND (blastp mode) with a minimum coverage of 50% for both query and subject sequences. Additionally, all 16S rRNA gene sequences were identified using barrnap (v0.9). Binning of the assemblies was performed using MetaBat2 (v2.12.1) with default settings (min contig length ≥ 1500 bp).^[^ ^64^ ^]^ Taxonomic assignment of the MAGs was performed using GTDB‐Tk based on the GTDB r207 database.^[^ ^2^ ^]^ The completeness and contamination of MAGs were determined using CheckM (v1.1.3) with lineage‐specific workflow and default parameters.^[^ ^65^ ^]^ Quality assessment of Patescibacteriota MAGs was performed using CheckM with a custom set of 43 marker genes specific to Patescibacteriota.^[^ ^1^ ^]^

### OTU Clustering and Taxonomy Assignment

To facilitate comparative analyses, rpS3 protein sequences were clustered, 16S rRNA gene sequences, and MAGs. The rpS3 and 16S rRNA gene sequences were clustered into OTUs at 97% identity using USEARCH (v7.0.1090) (‐sort length ‐id 0.97 ‐maxrejects 0 ‐maxaccepts 0).^[^ ^66^ ^]^ Only rpS3 OTUs were retained with sequences longer than 200 amino acids and 16S rRNA OTUs longer than 1,000 bp. All bacterial MAGs with completeness greater than 50% and contamination less than 10% were deduplicated into gOTUs using dRep (v3.2.2) with thresholds of 95% average nucleotide identity (ANI) and 30% coverage overlap.^[^ ^67^ ^]^

For taxonomy assignment of cprOTUs, a phylogenetic analysis incorporating both cprOTUs and reference sequences was constructed from the rpS3 database. Specifically, DIAMOND was first used to compare rpS3 OTU sequences against the rpS3 database to distinguish cprOTUs from other bOTUs, requiring at least 50% sequence coverage and 30% identity. Subsequently, the identified cprOTUs and aligned them with reference sequences were extracted from the rpS3 database. Multiple sequence alignments were generated using MAFFT and trimmed with Gblocks. A phylogenetic tree was then constructed using FastTree under the WAG model to determine the taxonomy of the cprOTUs. Taxonomic annotation for the 16S rRNA OTUs was performed using the SILVA SSU RefNR99 138 database classifier in QIIME2 (v2022.8.0).^[^ ^68^ ^]^

### Community Analysis

To estimate the relative abundance of Patescibacteriota, Salmon (v1. 3.0) was employed to generate an OTU table by using the nucleotide sequences of rpS3 OTUs.^[^ ^69^ ^]^ To assess the diversity of Patescibacteriota in metagenomic samples, alpha diversity (Shannon index and PD index) and beta diversity (weighted Unifrac distances) using the “microeco” package in R were calculated, based on the relative abundance of rpS3 OTUs (TPM). Principal coordinates analysis (PCoA) based on weighted UniFrac distances and non‐parametric multivariate analysis of variance (ADONIS) were used to examine the microbial community dissimilarity between diverse habitats. Additionally, a linear discriminant analysis effect size (LEfSe) analysis was performed to identify Patescibacteriota biomarkers at the family level across different environments.

### Network Analysis

To capture a comprehensive set of Patescibacteriota‐bacterial correlations and enhance the robustness of the inferred networks, random resampling and bootstrap strategies were employed.^[^ ^37^, ^70^ ^]^ Specifically, for each habitat, 10 samples were randomly selected with replacement to generate a data subset for network construction, repeating this process 999 times. In each data subset, OTUs were filtered by excluding those that appeared fewer than three times across the 10 samples, but abundance filtering was not applied to prevent the loss of low‐abundance cprOTUs. Network analysis was performed using R package CoNetinR (). Spearman correlation was used to assess the network associations, and correlations with *P*‐values greater than 0.05 were set to zero to exclude non‐significant associations. After completing the 999 iterations of random sampling, the correlation matrices from each iteration were aggregated, and the median correlation coefficient was used to represent the final correlation between each pair of OTUs. To ensure that the resulting ecological networks adhered to ecological principles, random matrix theory (RMT) was applied to the correlation coefficients, retaining only those with weights above the threshold. The final co‐occurrence networks of each habitat were further analyzed for interaction diversity using the Shannon diversity index, calculated using the network‐level function from the R package bipartite (https://github.com/biometry/bipartite).

### Metabolism Prediction of Patescibacteriota and Bacteria in a Network

To investigate the potential roles of bacteria co‐occurring with Patescibacteriota in biogeochemical cycling and virulence factor secretion, representative genomes corresponding to bOTUs at the genus level were collected. Bacterial MAGs for 13 genera were downloaded from GTDB (minimum n = 51, maximum n = 589) to evaluate the range of rpS3 identity within each genus. The mean rpS3 identity within each genus was found to be above 85% (ranging from 85.05% to 98.35%).

The representative genomes of bOTUs associated with cprOTUs in the network, representing potential hosts of Patescibacteriota, were identified by searching bOTU sequences against the rpS3 database, NR database, and rpS3 sequences from locally assembled bacterial MAGs, requiring a minimum of 50% query and subject sequence coverage and at least 85% identity. Bin quality was assessed using CheckM, retaining only MAGs with completeness above 50% and contamination below 10%. Protein prediction was performed using Prodigal v2.6.3, followed by functional annotation through alignment against KEGG and PHI‐base databases with at least 50% sequence coverage and 30% identity. Based on genome completeness, MAGs were considered to possess a specific metabolic capability only if at least 70% of the essential pathway genes were present (Table S7, Supporting Information).

To elucidate potential interaction mechanisms between Patescibacteriota and their hosts, representative genomes were identified for Patescibacteriota and host bacteria at the OTU level, requiring rpS3 sequence identity of at least 97% to the corresponding rpS3 OTU. Genome quality was assessed using CheckM, retaining only MAGs with completeness above 50% and contamination below 10%. For comprehensive metabolic potential assessment, ORFs of all genomes were predicted using Prodigal v2.6.3 and annotated against KEGG, COG,^[^ ^71^ ^]^ NR, and Pfam databases.^[^ ^72^ ^]^ All functional annotations required at least 50% sequence coverage and 30% identity.

### Sequence and Phylogenetic Analysis of Nitrite Reductase and Nitric Oxide Reductase

All Patescibacteriota MAGs from GTDB r207 and MAGs corresponding to cprOTUs were systematically screened for the presence of nitrite reductase (*nirK*) and nitric oxide reductase (*norB*). Protein sequences were predicted using Prodigal, and putative NirK and NorB proteins were identified through alignment against the KEGG database, requiring a minimum 50% coverage for both query and subject sequences. The identified NirK and NorB proteins of Patescibacteriota, along with reference sequences, were subjected to phylogenetic analysis. Multiple sequence alignments were performed using MAFFT and Gblocks. Phylogenetic trees for both proteins were constructed using IQ‐TREE (v2.3.5) with 1000 ultrafast bootstrap replicates, employing the LG+I+R7 evolutionary model for NirK and the LG+F+G4 model for NorB, as determined by ModelFinder.^[^ ^73^ ^]^

To characterize the copper/quinol‐binding and catalytic sites of NirK and NorB, detailed multiple sequence alignments of the assembled NirK and NorB sequences with reference sequences using MAFFT with default parameters were conducted. The alignment files were manually refined to ensure accuracy, and the results were visualized using BioEdit (v7.0.9.0).^[^ ^74^ ^]^ The quality and reliability of the predicted structural models were rigorously assessed based on the estimated TM‐scores provided by the I‐TASSER output,^[^ ^75^ ^]^ with higher scores indicating greater confidence in the predicted structures.

### Statistical Analyses

All statistical analyses were performed using R v4.3.1. Shapiro‐Wilk and Levene's tests were used to assess data normality and homogeneity of variance. As the relative abundance and alpha diversity data of the Patescibacteriota community did not satisfy assumptions of normal distribution and homoscedasticity (P > 0.05), the non‐parametric Kruskal‐Wallis test was employed to evaluate overall differences among habitat groups (P < 0.05). Games‐Howell post‐hoc tests were then performed for multiple comparisons between groups to identify specific between‐group differences in abundance and diversity metrics. To investigate the relationship between the network interaction diversity index and alpha diversity index (Shannon and PD index) across different habitats, ordinary least squares (OLS) regression was employed to estimate linear relationships.

## Conflict of Interest

The authors declare no conflict of interest.

## Author Contributions

Y. Ji, X. Liu, and S. Zhao are the co‐first authors and contributed equally to this article. The study was designed by Y.J., X.L., S. Z., and B.W. The metagenomic samples were collected by Sihan, Y.Y., and P.Z. The bioinformatic analysis was performed by Y.J., X.L., S. Z., Y.S., and B.W. The research results were discussed by Y.J., X.L., W.Q., G.Z., and B.W. The Original draft was written by B.W., X.L., S. Z., and Y.J. The manuscript was reviewed and edited by Y.J., X.L., S. Z., G.Z., Y.Z., J.J., Y.G., and B.W. Funding acquisition and supervision were by B.W. and Y.G.

## Supporting information



Supporting Information

Supplemental Table 1

Supplemental Table 2

Supplemental Table 3

Supplemental Table 4

Supplemental Table 5

Supplemental Table 6

Supplemental Table 7

Supplemental Table 8

## Data Availability

The data that support the findings of this study are available in the supplementary material of this article.
