# Supplementary material for: Unveiling Global Diversity of Patescibacteriota and Functional Interactions with Host Microbes
Source: Adv Sci (Weinh). 2025 Dec 12;13(11):e09416. doi: 10.1002/advs.202509416 (PMC12931201; doi:10.1002/advs.202509416)
Supplement: Supplementary file 1 — Supporting Information [file ADVS-13-e09416-s004.docx]

Supporting Information

**Unveiling Global Diversity of Patescibacteriota and Functional Interactions with Host Microbes**

*Yanhan Ji,^#^ Xu Liu,^#^ Shuai Zhao,^#^ Sihan Zhou, Yufan Yang, Ping Zhang, Yu Shi, Wei Qin, Guibing Zhu, Yongguan Zhu, Yanzheng Gao,* Jiandong Jiang,* Baozhan Wang**

Y. Ji, X. Liu, S. Zhou, Y. Yang, P. Zhang, Y. Gao, J. Jinag, B. Wang

Key Laboratory of Agricultural and Environmental Microbiology, Ministry of Agriculture and Rural Affairs, Department of Microbiology, College of Life Sciences, Nanjing Agricultural University, Nanjing, Jiangsu 210095, China

E-mail: [bzwang@njau.edu.cn](mailto:bzwang@njau.edu.cn); [jiang_jjd@njau.edu.cn](mailto:jiang_jjd@njau.edu.cn); [gaoyanzheng@njau.edu.cn](mailto:gaoyanzheng@njau.edu.cn)

B. Wang

State Key Laboratory of Herbage Improvement and Grassland Agro-ecosystems, Centre for Grassland Microbiome, College of Pastoral Agriculture Science and Technology, Lanzhou University, Lanzhou, Gansu 730020, China

S. Zhao

State Key Laboratory of Desert and Oasis Ecology, Xinjiang Institute of Ecology and Geography, Chinese Academy of Sciences, Urumqi 830011, China

Y. Shi

State Key Laboratory of Crop Stress Adaptation and Improvement, School of Life Sciences, Henan University, Kaifeng 475004, China

W. Qin

School of Biological Sciences, and Institute for Environmental Genomics, University of Oklahoma, Norman, OK 73072, USA

G. Zhu

Research Center for Eco-Environmental Sciences, Chinese Academy of Sciences, Beijing 100085, China

Y. Zhu

State Key Laboratory of Urban and Regional Ecology, Research Centre for Eco-Environmental Sciences, Chinese Academy of Sciences, Beijing 100085, China

**^#^**These three authors contributed equally to this article.

***Corresponding authors:** Baozhan Wang ([bzwang@njau.edu.cn](mailto:bzwang@njau.edu.cn)), Jiandong Jiang ([jiang_jjd@njau.edu.cn](mailto:jiang_jjd@njau.edu.cn)), Yanzheng Gao ([gaoyanzheng@njau.edu.cn](mailto:gaoyanzheng@njau.edu.cn)).

**Keywords**: Patescibacteriota, CPR, Episymbionts, Ribosomal protein S3, Metabolic complementation, Nitrite detoxification

**

**

**Figure S1. Summary of Patescibacteriota statistics from GTDB R207.** (**a**) Overview of Patescibacteriota genome statistics, including the total number of CPR genomes, the number of Patescibacteriota 16S rRNA gene sequences, the number of CPR rpS3 sequences, and the count of Patescibacteriota 16S rRNA gene sequences containing insertions. (**b**) Analysis of insertions in Patescibacteriota 16S rRNA gene sequences, showing the number of sequences with insertions and the number of insertions per sequence. (**c**) Length distribution of 16S rRNA gene sequences with insertions, the same sequences with insertions removed, rpS3 gene sequences, and the insertion sequences themselves.





**Figure S2. Phylogeny of Patescibacteriota bacteria.** (**a, b, c**) Phylogenetic trees of 4,645 Patescibacteriota from GTDB R207, constructed using concatenated phylogenetic marker amino acid sequences from GTDB-Tk v2.1.1, the 16S rRNA gene nucleotide sequences, and rpS3 amino acid sequences, respectively. Hollow circles and squares in the phylogenetic tree of concatenated marker genes indicate clades that are absent in the 16S rRNA gene and rpS3 phylogenetic trees, respectively. The clade marked by a blue circle in the concatenated marker genes tree splits into two distinct clades in the 16S rRNA phylogenetic tree. Clades marked by colored squares in the concatenated marker genes tree cluster into two independent clades in the rpS3 phylogenetic tree.





**Figure S3. Comparison of unique 16S rRNA sequences, MAGs, and rpS3 sequences counts across eight different habitats.** Bar plots with individual data points show the mean number of unique sequences (± standard error) assembled from metagenomic data for each habitat. Significant differences between number of unique sequence types within each habitat were determined using t-test with *P*-value **P* < 0.05, ***P* < 0.01, ****P* < 0.001.





**Figure S4**. **Rarefaction curves at the genus level for Patescibacteriota.** Rarefaction analyses were performed to evaluate the sampling effort and genus-level diversity of Patescibacteriota in metagenomic samples from freshwater (n = 62), groundwater (n = 22), human body (n = 53), marine (n = 48), plant-associated (n = 36), saline lake (n = 60), soil (n = 28), and wastewater treatment plants (WWTPs, n = 30). The y-axis indicates the number of accumulated genera, and the x-axis shows the number of samples.





**Figure S5. Identification and distribution of Patescibacteriota family-level biomarkers across different habitats.** (**a**) Biomarkers predicted by LEfSe analysis for Patescibacteriota taxa at the family level in different habitats. (**b**) Heatmap illustrating the relative abundances of these Patescibacteriota families across samples.





**Figure S6. Phylogenetic representation of network analyses across eight distinct habitats.** Network analyses were conducted separately for eight different habitats, focusing exclusively on interactions between Patescibacteriota taxa and other bacteria. Nodes in each network were mapped onto branches of a phylogenetic tree in iTOL and arranged to form an inner circular tree. Connections between branches represent network associations: blue lines indicate positive associations, and red lines indicate negative associations. Branch colors represent taxonomic groups, with Patescibacteriota shown in purple and other bacteria in green.





**Figure S7. The relationship of alpha diversity index and network interaction diversity index.** (**a**) Linear regression analysis showing the correlation between Patescibacteriota Shannon diversity index and interaction diversity index. (**b**) Linear regression analysis showing the correlation between phylogenetic diversity (PD) index and interaction diversity index. The solid line represents the fitted linear regression, and the shaded area indicates the 95% confidence interval. *R*², *r* and *P*-values are shown for each correlation.

**

**

**Figure S8. Phylogenetic tree of cprOTUs associated with Desulfobacterota or *Nitrospira* in the network.** Different background colors represent distinct Patescibacteriota classes. Symbols on the tree indicate metabolic potential in corresponding genomes: yellow diamonds represent the presence of *nirK* gene encoding nitrite reductase, while orange circles indicate the presence of *norB* gene encoding nitric oxide reductase. cprOTUs are colored consistently with these symbols to suggest potential metabolic capabilities. The phylogenetic tree was conducted using IQ-TREE with Q.yeast+I+G4 model.

**

**

**Figure S9. Identification and structural prediction of NirK in Patescibacteriota.** (**a**) Genomic context of contigs encoding the NirK gene within Patescibacteriota representative genomes, showing surrounding genes, most of which originate from Patescibacteriota. (**b**) Multiple sequence alignment of CPR NirK with other reference sequences, highlighting conserved copper-binding motifs (T1Cu in green background and T2Cu in blue background) and active site residues (Asp and His, essential for nitrite-reducing activity, in orange background). (**c**) Predicted tertiary structure of Patescibacteriota NirK using I-TASSER, illustrating the protein's structural features.





**Figure S10. Phylogenetic tree of *Nitrospira* genomes based on concatenated phylogenetic marker genes.** The tree is colored to distinguish canonical NOB (green) and comammox (yellow). Colored arrows on the tree indicate genomes with high similarity (>97%) between their rpS3 sequences and the bOTUs in the network. The phylogenetic tree was conducted using IQ-TREE with JTT+F+R7 model.





**Figure S11. Identification and structural prediction of NorB in Patescibacteriota.** (**a**) Genomic context of contigs encoding the NorB gene within Patescibacteriota representative genomes, displaying surrounding genes, most of which are derived from Patescibacteriota. (**b**) Multiple sequence alignment of Patescibacteriota NorB with reference sequences, highlighting strongly conserved residues in red background. (**c**) Predicted crystal structure of Patescibacteriota NorB generated using I-TASSER, illustrating key structural features.





**Figure S12. Schematic summary of key Patescibacteriota–host interactions. ​**The left panel shows a co-occurrence network, where green nodes represent Patescibacteriota lineages (class-level) and red nodes indicate their putative hosts (phylum-level), highlighting the taxonomic breadth of Patescibacteriota–host associations. The right panel presents two representative interaction models derived from genomic annotations: Patescibacteriota may mitigate nitric oxide toxicity for CMX (*UBA1550*) and assist SRB in resisting nitrite stress (*C7867-001* and *OLB19*).
